# Supplementary material for: DL4-μbeads induce T cell lineage differentiation from stem cells in a stromal cell-free system
Source: Nat Commun. 2021 Aug 18;12:5023. doi: 10.1038/s41467-021-25245-8 (PMC8373879; doi:10.1038/s41467-021-25245-8)
Supplement: Supplementary file 1 — Supplementary Information [file 41467_2021_25245_MOESM1_ESM.pdf]

Supplementary Figures 1-8

Supplementary Tables 1-3

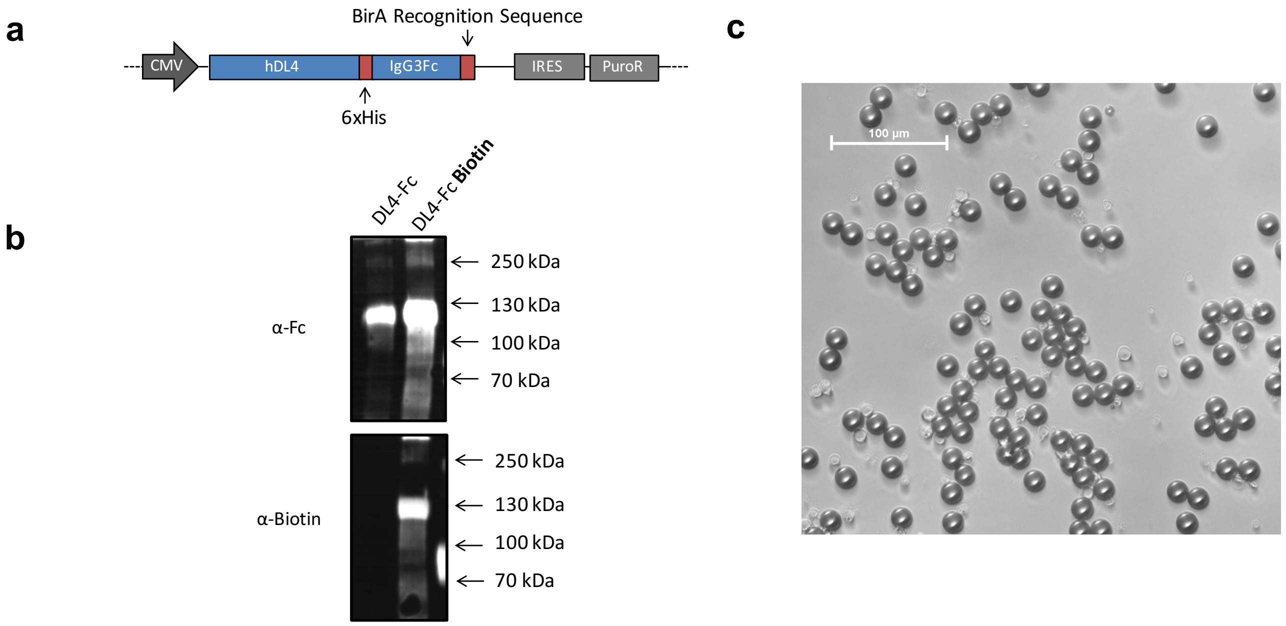

Supplementary Figure 1: Site-specific biotinylation of DL4-Fc.

a, Biotinylation of DL4-Fc. DL4-Fc fusion construct was redesigned to include a BirA recognition sequence (AviTag^{TM}) at its C-terminus, to which a biotin moiety may be conjugated by the enzyme BirA. **b,** Western blot analysis of chemically biotinylated DL4-Fc using anti-human IgG (top) and anti-biotin (bottom) under reducing conditions. **c,** Microscopy of DL4-µbead interaction with mouse LSKs. 100 mm scale bar is included in the top left of the image. Western blot and microscopy images displayed are representative of 2 independent experiments.

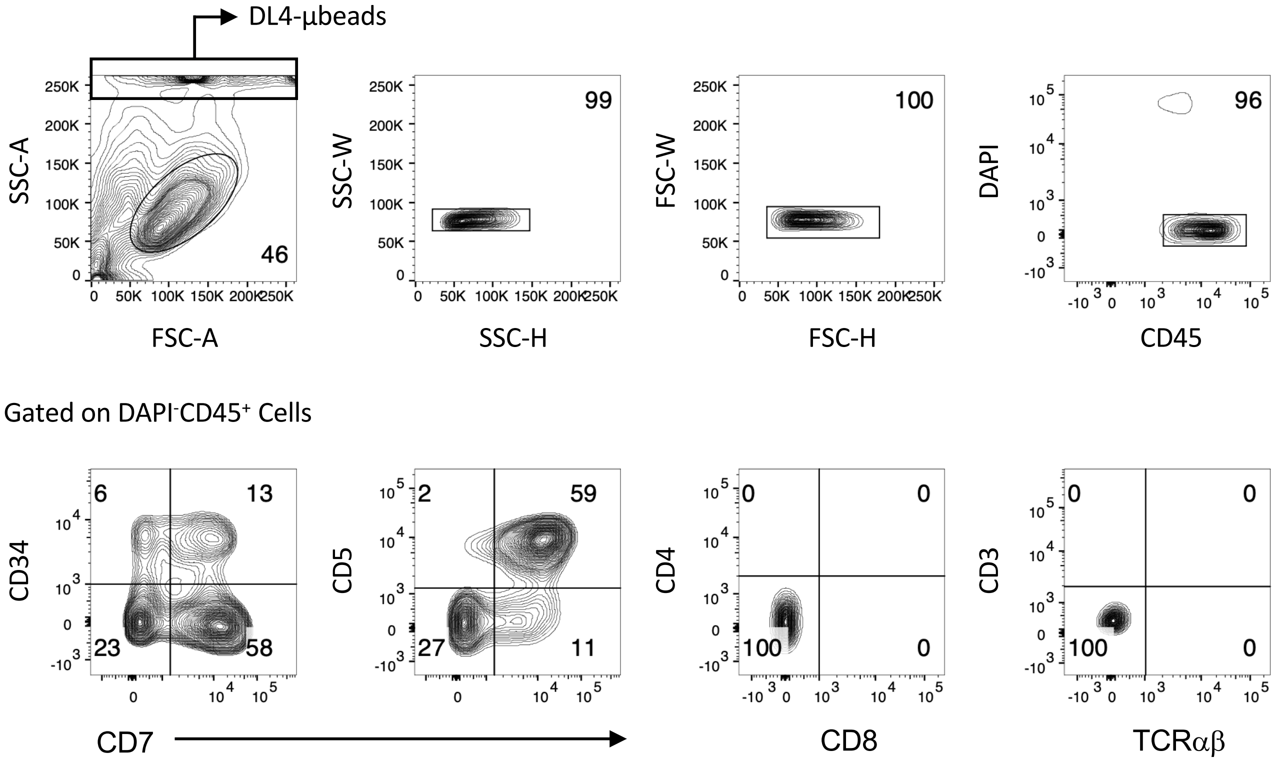

Supplementary Figure 2: Gating strategy for human T-lineage analysis

Representative flow cytometry plots of human CB-CD34$^+$ cells cultured with DL4-µbeads for 14 days. Identical gating strategy was used for all other human samples (CB, mPB and PSC). Side scatter (SSC) / forward scatter (FSC) plots were used to identify lymphocyte populations and single cell events. Cells were further gated on CD45 expression and for the lack of 4′,6-diamidino-2-phenylindole (DAPI) uptake. Gated cells were analyzed for the expression of indicated surface markers.

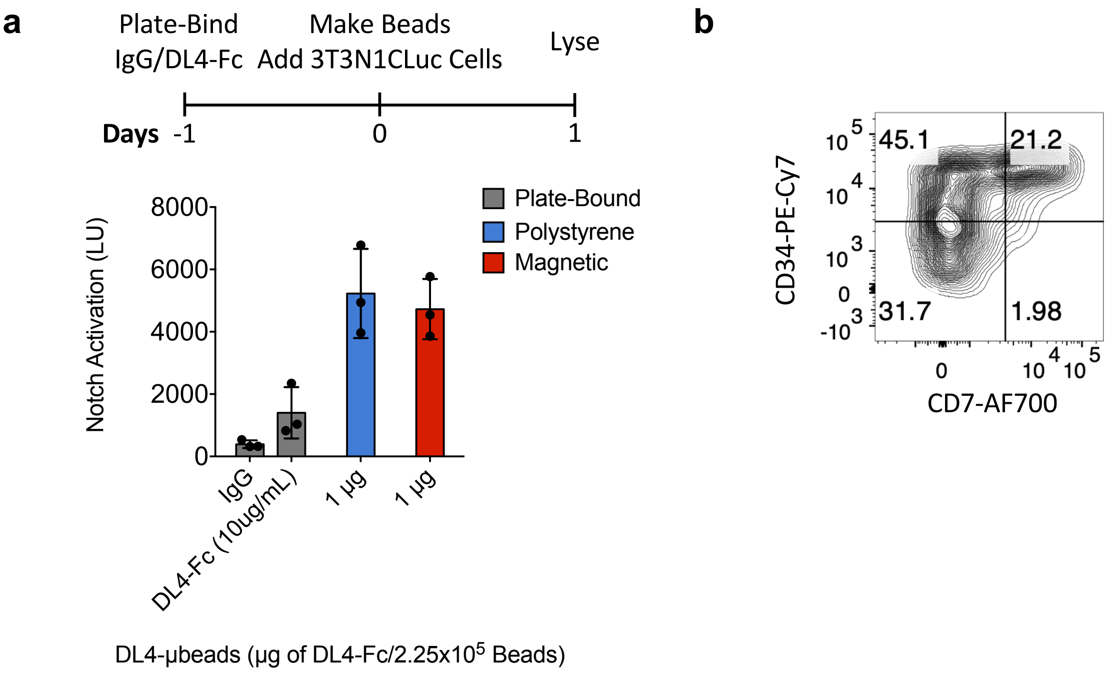

Supplementary Figure 3: Evaluation of magnetic DL4-μbeads

a, Evaluation of bead core composition on Notch activation and cell separation. 2 x 10^4 3T3N1Cluc cells were incubated on plates pretreated with hIgG as negative control or with DL4-Fc at 10 mg/mL. 25mm polystyrene and magnetic streptavidin-coated μbeads were incubated overnight with 3T3N1Cluc cells. Plate-bound IgG and DL4-Fc were included as controls. 24 h after plating, the cells were lysed and analyzed for luciferase activity. Data represent means ± s.d. of *n* = *3* independent experiments. **b,** Representative flow cytometry plots of human cord blood-derived $CD34^+$ cells cultured for 7 with DL4-μbeads in serum-free media supplemented with SCF, IL-7 and Flt3-L. Cells were harvested, magnetically separated using autoMACS-pro cells sorter (Miltenyi Biotec, Auburn, CA) and analyzed for the surface expression of CD34, CD5, and CD7 using flow cytometry.

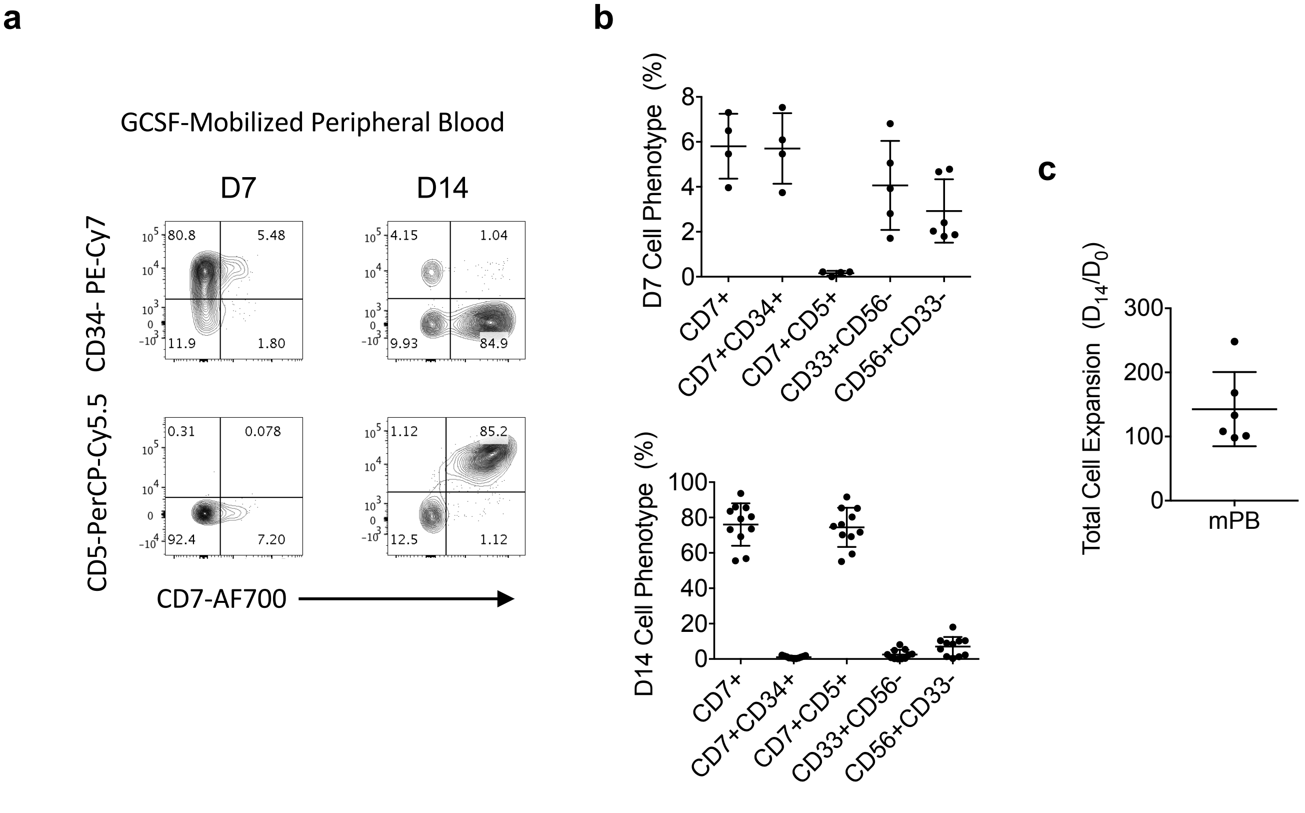

Supplementary Figure 4: Human proT cell development from GCSF-mobilized CD34$^+$ cells.

a, Representative flow cytometry plots of GCSF-mobilized CD34$^+$ cells cultured for 7 and 14 days with DL4-μbeads in serum-free media supplemented with SCF, IL-7 and Flt3-L. Cells were harvested and analyzed for the surface expression of CD34, CD5, and CD7 using flow cytometry. **b,** Frequencies of the indicated proT-cell phenotypes after 7 days or 14 days. **c,** Total cell expansion on D14, normalized to input day 0 CD34$^+$ CB-derived CD34$^+$ after culture with DL4-μbeads (n = 6). Data represent means ± s.d. of n independent experiments.

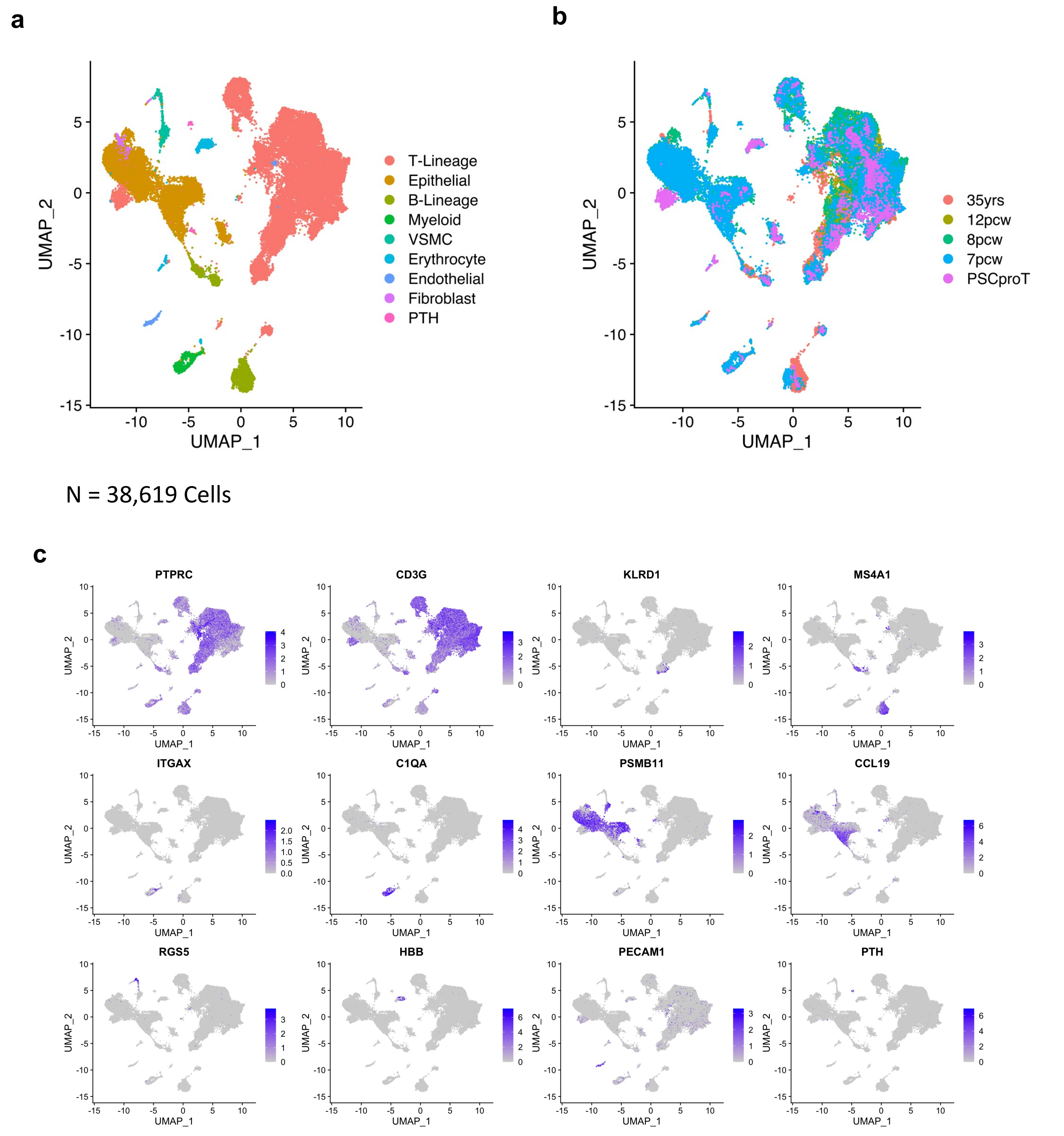

Supplementary Figure 5: Cellular composition of integrated human thymus datasets

a, UMAP visualization of integrated and batch-corrected sequencing data from D7 PSC-proT cells and primary human thymus cells (ArrayExpress: E-MTAB-8581) colored by cell type. Sequencing data from a total of 38,619 cells were integrated and batch-corrected, and differential expression of genes between clusters were used to identify and annotate hematopoietic and thymic stromal cells. **b,** Same UMAP plot colored by age groups, indicated by post-conception weeks (pcw) or postnatal years (yrs). **c,** UMAP visualization of the expression of curated feature genes for cell cluster identification.

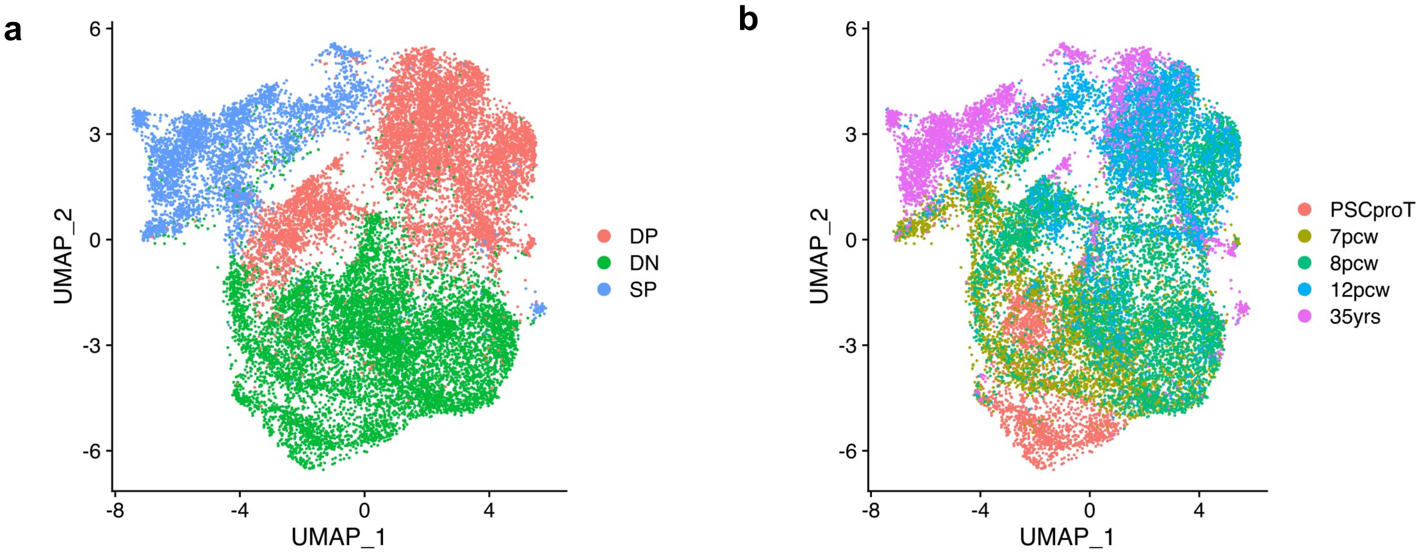

N = 21,439 Cells

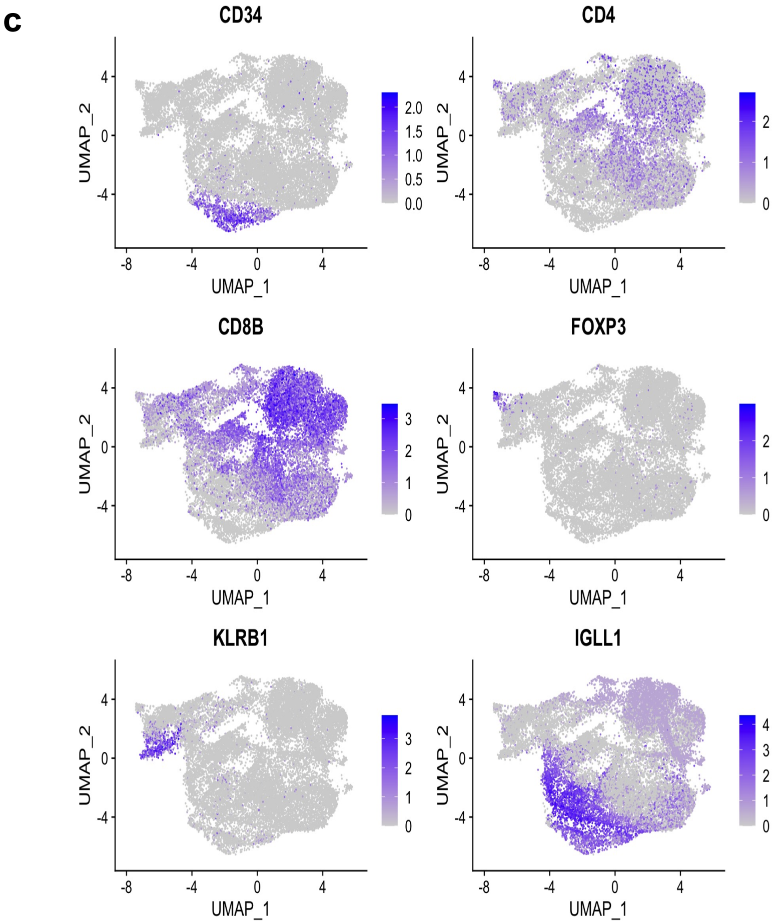

Supplementary Figure 6: UMAP visualization of T-lineage subset of integrated scRNA-seq data

a, UMAP visualization of integrated and batch-corrected sequencing data from PSC-proT cells and primary human thymocytes (ArrayExpress: E-MTAB-8581) colored by T-lineage subset. Canonical T-lineage marker genes and recently defined developmental markers (*ST18* for DN, *AQP3* for DP and *TOX2* for DP-to-SP transition) were used to identify subset. **b,** Same UMAP plot colored by age groups, indicated by post-conception weeks (pcw) or postnatal years (yrs). **c,** UMAP visualization of the expression of curated feature genes for cell cluster identification.

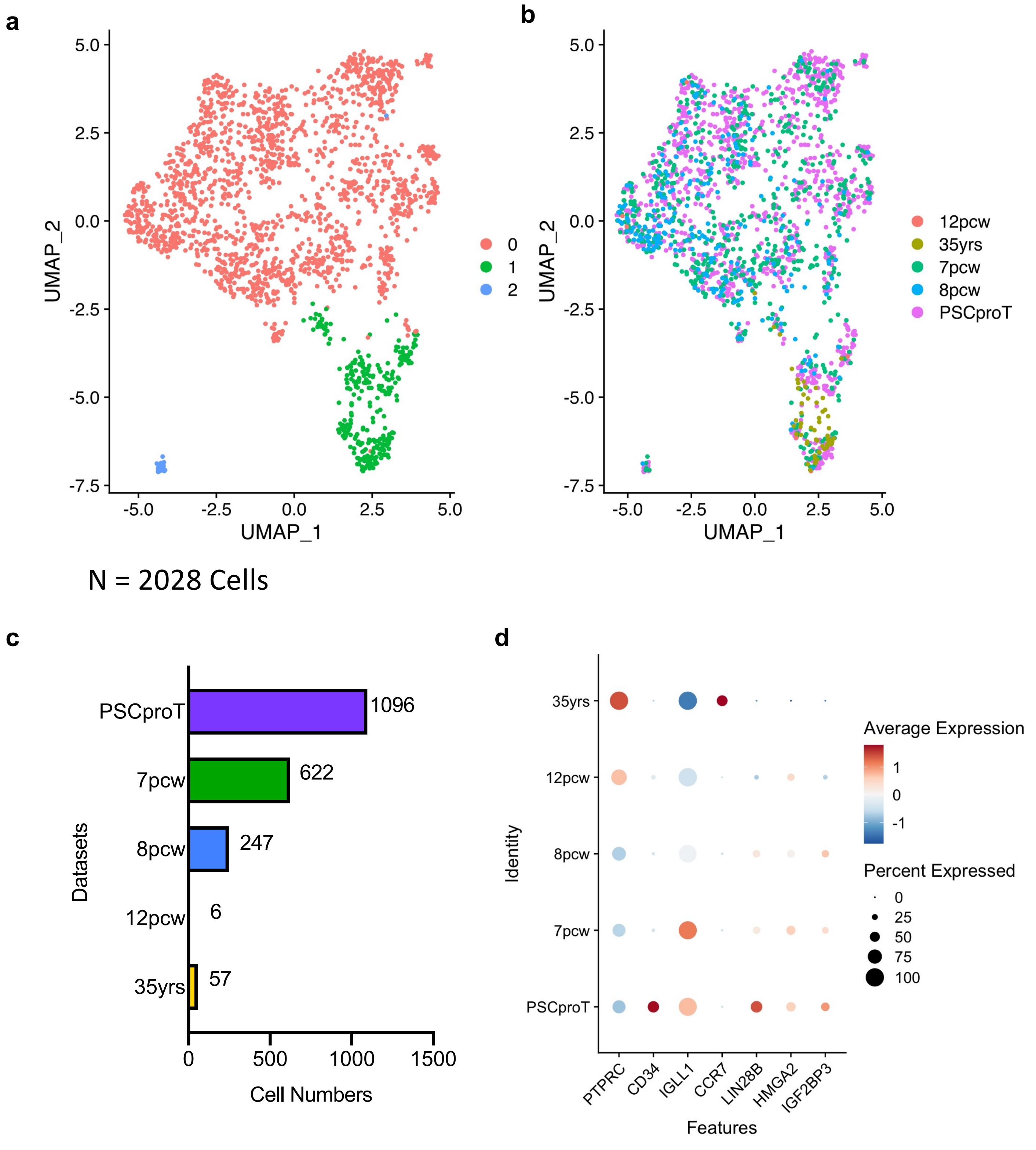

Supplementary Figure 7: DN subset of integrated scRNA-seq data

a, Cluster analysis on a subset of integrated single-cell RNA seq data based on the lack of expression of CD4, CD8, AQP3, TOX2, CD27. **b,** UMAP visualization of DN subset colored by age groups, indicated by post-conception weeks (pcw) or postnatal years (yrs). **c,** Cell numbers of each dataset within the DN subset. **d,** Dot plot for expression of curated marker genes.

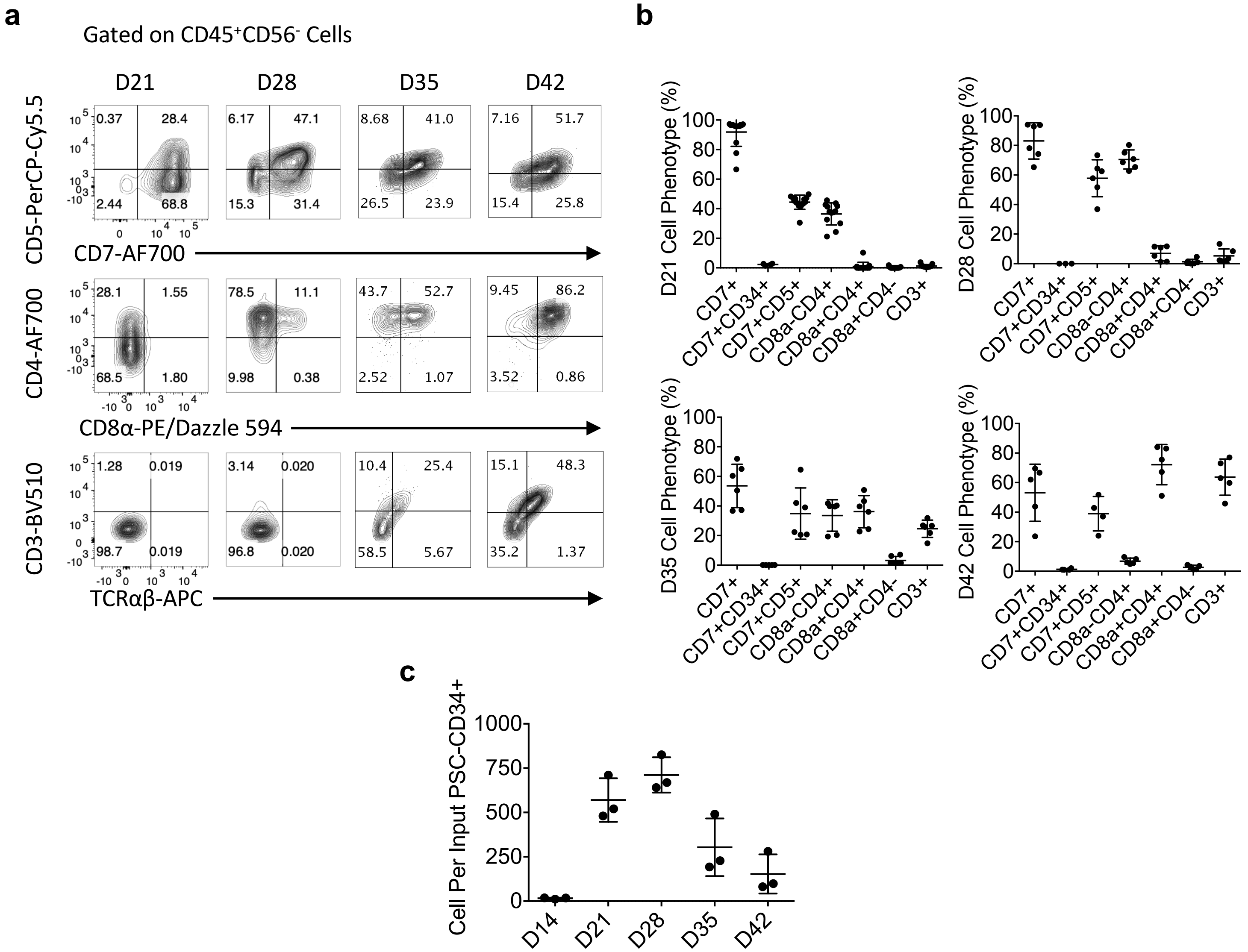

Supplementary Figure 8: T cell development from human pluripotent stem cells using DL4-mbeads

a, Representative flow cytometry plots of human iPSC-derived CD34+ cells cultured for up to 42 days. IPS11 cell line was used to generate all data. Cultures were subjected to weekly media changes and re-addition of DL4-mbeads. Cells were harvested and analyzed for the surface expression of CD5, CD7, CD4, CD8, TCRab and CD3 by flow cytometry. **b,** Frequencies of the indicated T-lineage phenotypes at various time points (D21: *n* = 11, D28: *n* = 6, D35: *n* = 6, D28: *n* = 5). **c,** Total cell expansion normalized to input day 0 iPSC-CD34+ cells after culture with DL4-μbeads (*n* = 3). Data represent means ± s.d. of *n* independent experiments.

Supplementary Table 1. T Cell Progenitor Frequency Analysis of CD34^{+} Cells From Multiple Sources of HSPCs

HSPC[a]	Progenitor Frequency^{-1} (95% Confidence Limits)[b]
CB	15 (8-28)
GCSF-mPB	172 (81-366)
iPSC	1341 (923-1949)

[a]MACS-enriched CD34^{+} cells were placed in limited numbers in wells of a 96-well plate containing DL4-µbeads and cultured for 14 days before harvesting for flow cytometric analysis

[b]Individual wells were scored for the presence of T cell progenitors based on CD45^{+}CD7^{+}CD56^{-}. Statistical analysis was performed via the method of maximum likelihood applied to the Poisson model[47].

Supplementary Table 2. T Cell Progenitor Frequency Analysis of CB-CD34^{+} Cells Cultured on Plate-Bound DL4-Fc vs DL4-μbeads

HSPC[a]	Progenitor Frequency^{-1} (95% Confidence Limits)[b]
PB (3 μg/mL)	17 (10-28)
PB (9 μg/mL)	16 (10-25)
DL4-μbeads	9 (6-15)

[a]MACS-enriched CD34^{+} cells were placed in limited numbers in wells of a 96-well plate containing either plate-bound (PB) DL4-Fc or DL4-μbeads and cultured for 7 days before harvesting for flow cytometric analysis

[b]Individual wells were scored for the presence of T cell progenitors based on CD45^{+}CD34^{+}CD7^{+}. Statistical analysis was performed via the method of maximum likelihood applied to the Poisson model[47].

Supplementary Table 3. Antibodies used in this study

Antibody	Clone	Fluorophore	Supplier	Catalog Number
CD45	HI30	APC/Cy7	BioLegend	304014
CD34	581	PE/Cy7	BioLegend	343516
CD34	563	PE	BioLegend	550761
CD7	6B7	FITC	BioLegend	343104
CD5	UCHT2	PerCP/Cy5.5	BioLegend	300620
CD4	RPA-T4	AF700	BioLegend	300526
CD8α	RPA-T8	PE/Dazzle 594	BioLegend	301058
TCRαβ	IP26	APC	BioLegend	306718
CD3	OKT3	BV510	BioLegend	317332
CD56	MEM-188	PE	BioLegend	304606
CD184	12G5	PerCP-eFluor 710	eBioscience	46-9999-42
CD73	AD2	APC	eBioscience	17-0739-42
IFNγ	B27	PerCP/Cy5.5	BioLegend	506527
TNFα	MAb11	PE-Cy7	BioLegend	502930
CD90	30-H12	PE	eBioscience	12-0903-83
CD25	PC61	APC	BioLegend	557658
CD11b	M1/70	FITC	eBioscience	11-0112-82
CD19	6D5	PE/Cy7	BioLegend	115519
CD4	GK1.5	FITC	BioLegend	100405
CD8	53-6.7	PE	BioLegend	100707
CD3	145-2C11	PE/Cy7	BioLegend	100320
TCRβ	H57-597	APC	BioLegend	109212
