# Supplementary material for: DL4-μbeads induce T cell lineage differentiation from stem cells in a stromal cell-free system
Source: Nat Commun. 2021 Aug 18;12:5023. doi: 10.1038/s41467-021-25245-8 (PMC8373879; doi:10.1038/s41467-021-25245-8)
Supplement: Supplementary file 3 — Reporting Summary [file 41467_2021_25245_MOESM3_ESM.pdf]

nature research

Corresponding author(s): Juan Carlos Zuniga-Pflucker

Last updated by author(s): May 31, 2021

Reporting Summary

Nature Research wishes to improve the reproducibility of the work that we publish. This form provides structure for consistency and transparency in reporting. For further information on Nature Research policies, see Authors & Referees and the Editorial Policy Checklist .

Statistics

For all statistical analyses, confirm that the following items are present in the figure legend, table legend, main text, or Methods section.

n/a	Confirmed	
☐	☒	The exact sample size (*n*) for each experimental group/condition, given as a discrete number and unit of measurement
☐	☒	A statement on whether measurements were taken from distinct samples or whether the same sample was measured repeatedly
☐	☒	The statistical test(s) used AND whether they are one- or two-sided<br>*Only common tests should be described solely by name; describe more complex techniques in the Methods section.*
☒	☐	A description of all covariates tested
☒	☐	A description of any assumptions or corrections, such as tests of normality and adjustment for multiple comparisons
☐	☒	A full description of the statistical parameters including central tendency (e.g. means) or other basic estimates (e.g. regression coefficient) AND variation (e.g. standard deviation) or associated estimates of uncertainty (e.g. confidence intervals)
☐	☒	For null hypothesis testing, the test statistic (e.g. *F*, *t*, *r*) with confidence intervals, effect sizes, degrees of freedom and *P* value noted<br>*Give P values as exact values whenever suitable.*
☒	☐	For Bayesian analysis, information on the choice of priors and Markov chain Monte Carlo settings
☒	☐	For hierarchical and complex designs, identification of the appropriate level for tests and full reporting of outcomes
☒	☐	Estimates of effect sizes (e.g. Cohen's *d*, Pearson's *r*), indicating how they were calculated

Our web collection on statistics for biologists contains articles on many of the points above.

Software and code

Policy information about availability of computer code

Data collection	Flow cytometry data was obtained using BD Biosciences LSR II. Single-cell RNA sequencing was performed using 10X Genomics.
Data analysis	Flow cytometry data was analyzed using FlowJo 9.9.6. Statistical analysis was performed using Prism 6 software. Progenitor frequencies were determined by the method of maximum likelihood applied to the Poisson model using the following software: http://bioinf.wehi.edu.au/software/elda/. Single-cell RNA sequencing data was analyzed using R software 4.0.2 with the package Seurat (version 4.0).

For manuscripts utilizing custom algorithms or software that are central to the research but not yet described in published literature, software must be made available to editors/reviewers. We strongly encourage code deposition in a community repository (e.g. GitHub). See the Nature Research guidelines for submitting code & software for further information.

Data

Policy information about availability of data

All manuscripts must include a data availability statement. This statement should provide the following information, where applicable:

- Accession codes, unique identifiers, or web links for publicly available datasets
- A list of figures that have associated raw data
- A description of any restrictions on data availability

The data that support the findings of this study are available from the corresponding author upon request.
Accession codes for the single-cell RNA sequencing data are provided and deposited GEO under the accession number GSE169279.

Field-specific reporting

Please select the one below that is the best fit for your research. If you are not sure, read the appropriate sections before making your selection.

- [x] Life sciences
- [ ] Behavioural & social sciences
- [ ] Ecological, evolutionary & environmental sciences

For a reference copy of the document with all sections, see nature.com/documents/nr-reporting-summary-flat.pdf

Life sciences study design

All studies must disclose on these points even when the disclosure is negative.

Sample size	Sample size was determined according to standard practices in the field.
Data exclusions	No data were excluded in these studies.
Replication	All methods for each particular experiment were performed in a similar fashion, including the same materials used. Three to four independent experiments were performed (which were reliably reproduced).
Randomization	Mice were age matched (3-5 days neonatal) for each particular experiment.
Blinding	Scoring for LDA was performed in a blinded fashion.

Reporting for specific materials, systems and methods

We require information from authors about some types of materials, experimental systems and methods used in many studies. Here, indicate whether each material, system or method listed is relevant to your study. If you are not sure if a list item applies to your research, read the appropriate section before selecting a response.

Materials & experimental systems

n/a	Involved in the study	
☐	☒	Antibodies
☐	☒	Eukaryotic cell lines
☒	☐	Palaeontology
☐	☒	Animals and other organisms
☐	☒	Human research participants
☒	☐	Clinical data

Methods

n/a	Involved in the study	
☒	☐	ChIP-seq
☐	☒	Flow cytometry
☒	☐	MRI-based neuroimaging

Antibodies

Antibodies used

FITC anti-human CD7 Antibody (clone CD7-6B7) cat # 343104. Supplier: Biolegend (1:200)
PerCP/Cyanine5.5 anti-human CD5 Antibody (clone UCHT2) cat # 300620. Supplier: Biolegend (1:200)
PE/Cyanine7 anti-human CD34 Antibody (clone 581) cat # 343516. Supplier: Biolegend (1:200)
PE/Dazzle™ 594 anti-human CD8a Antibody (clone RPA-T8) cat # 301058. Supplier: Biolegend (1:200)
PE anti-human CD56 (NCAM) Antibody (clone MEM-188) cat # 304606. Supplier: Biolegend (1:200)
PE-Cyanine7 anti-human/non-human primate CD8b Antibody (clone SIDI8BEE) cat # 25-5273-42. Supplier: eBioscience™/ThermoFisher Scientific (1:200)
APC anti-human CD1a Antibody (clone HI149) cat # 300110. Supplier: Biolegend (1:200)
Alexa Fluor® 700 anti-human CD4 Antibody (clone RPA-T4) cat # 300526. Supplier: Biolegend (1:200)
APC/Cyanine7 anti-human CD45 Antibody (clone HI30) cat # 304014. Supplier: Biolegend (1:200)
Brilliant Violet 510™ anti-human CD3 Antibody (clone OKT3) cat # 317332. Supplier: Biolegend (1:200)
APC anti-human TCR α/β Antibody (clone IP26) cat # 306718. Supplier: Biolegend (1:200)
PerCP-eFluor 710 anti-human CD184 (CXCR4) Antibody (clone 12G5) cat # 46-9999-42. Supplier: eBioscience™/ThermoFisher Scientific (1:200)
APC anti-human CD73 Antibody (clone AD2) cat # 17-0739-42. Supplier: eBioscience™/ThermoFisher Scientific (1:200)
PE anti-human CD34 Antibody (clone 563) cat # 550761. Supplier: BD Biosciences (1:200)
PerCP/Cy5.5 anti-human IFNγ Antibody (clone B27) cat # 506527. Supplier: BioLegend (1:200)
PE-Cy7 anti-human TNFα Antibody (clone MAb11) cat # 502930. Supplier: BioLegend (1:200)
PE anti-mouse CD90 Antibody (clone 30-H12) cat # 12-0903-83. Supplier: eBioscience (1:400)
APC anti-mouse CD25 Antibody (clone PC61) cat # 557658. Supplier: BioLegend (1:400)
FITC anti-mouse CD11b Antibody (clone M1/70) cat # 11-0112-82. Supplier: eBioscience (1:400)
PE/Cy7 anti-mouse CD19 Antibody (clone 6D5) cat # 115519. Supplier: BioLegend (1:400)

	FITC anti-mouse CD4 Antibody (clone GK1.5) cat # 100405. Supplier: BioLegend (1:400)<br>PE anti-mouse CD8 Antibody (clone 53-6.7) cat # 100707. Supplier: BioLegend (1:200)<br>PE/Cy7 anti-mouse CD3 Antibody (clone 145-2C11) cat # 100320. Supplier: BioLegend (1:400)<br>APC anti-mouse TCRβ Antibody (clone H57-597) cat # 109212. Supplier: BioLegend (1:400)
Validation	All listed antibodies have been validated by the company indicated, and displayed in their material information for each product.

Eukaryotic cell lines

Policy information about cell lines

Cell line source(s)	Human iPS11 (Alstem Cell Advancements, Richmond, CA)<br>STiPS A3 (HSCI iPS Cell Core Facility, Boston, MA)<br>NIH3T3 (ATCC, Manassas, VA)<br>HEK-293T (ATCC, Manassas, VA)
Authentication	Cell lines sourced from ATCC (NIH3T3 and HEK-293T cells) were authenticated by STR profiling. Human IPS11 and STiPS A3 were not authenticated.
Mycoplasma contamination	Cell lines were not tested for mycoplasma contamination.
Commonly misidentified lines<br>(See ICLAC register)	No commonly misidentified cell lines were used in these studies.

Animals and other organisms

Policy information about studies involving animals; ARRIVE guidelines recommended for reporting animal research

Laboratory animals	NSG and C57BL/6 mice were purchased from Jackson Laboratory (Bar Harbor, ME), housed, bred and maintained in the Comparative Research Facility of the Sunnybrook Research Institute, under specific pathogen-free conditions. 3-5 day neonatal mice of both male and females were used.
Wild animals	The study did not involve wild animals.
Field-collected samples	This study did not involve field-collected samples.
Ethics oversight	All mice were bred and maintained in the Comparative Research Facility of the Sunnybrook Research Institute under specific pathogen-free conditions. All animal procedures were approved by the Sunnybrook Research Institute Animal Care Committee and performed in accordance with the committee's ethical standards.

Note that full information on the approval of the study protocol must also be provided in the manuscript.

Human research participants

Policy information about studies involving human research participants

Population characteristics	*Describe the covariate-relevant population characteristics of the human research participants (e.g. age, gender, genotypic information, past and current diagnosis and treatment categories). If you filled out the behavioural & social sciences study design questions and have nothing to add here, write "See above."*
Recruitment	*Describe how participants were recruited. Outline any potential self-selection bias or other biases that may be present and how these are likely to impact results.*
Ethics oversight	Human umbilical cord blood samples were from consenting mothers following delivery in accordance to approved guidelines established by the Research Ethics Board of Sunnybrook Health Sciences Centre.

Note that full information on the approval of the study protocol must also be provided in the manuscript.

Flow Cytometry

Plots

Confirm that:

- [x] The axis labels state the marker and fluorochrome used (e.g. CD4-FITC).
- [x] The axis scales are clearly visible. Include numbers along axes only for bottom left plot of group (a 'group' is an analysis of identical markers).
- [x] All plots are contour plots with outliers or pseudocolor plots.
- [x] A numerical value for number of cells or percentage (with statistics) is provided.

Methodology

Sample preparation	Bone marrow, thymus, and spleen samples were harvested and placed in alpha-mem media supplemented with 20% FBS. Bone marrow were crushed, while thymus and spleen were passed through 40um cell strainers for single cell preparations. Cells were then subsequently resuspended in Hanks' balanced salt solution containing 1% BSA with appropriate antibodies for staining. Cells were then washed and resuspended in 1% BSA HBSS containing DAPI prior to analysis.
Instrument	Flow cytometry data was obtained using BD Biosciences LSR II.
Software	Flow cytometry data was analyzed using FlowJo 9.9.6.
Cell population abundance	Purity of post-sort samples was determined by taking a fraction of the post-sort sample and running FACS analysis.
Gating strategy	All cells were first pre-gated on FSC/SSC and then on DAPI- for identification of live cells. Plots for precise gating strategies for any specific experiment can be provided upon request.

☒ Tick this box to confirm that a figure exemplifying the gating strategy is provided in the Supplementary Information.
